# Improving the Compliance of Massive Hemorrhage Protocols Through Education Is Associated with Patient Survival

**DOI:** 10.3390/jcm14134632

**Published:** 2025-06-30

**Authors:** Pilar Paniagua-Iglesias, Maria Dolores Rincón-Ferrari, Angel Candela-Toha, Maria Marcos-Jubilar, Marta Barquero-Lopez, Ignasi Gich-Saladich, Laura Medina-Marrero, Alba Bosch-Llobet, Daniela Garrido-Fleischmann, Jordi Ordoñez-Llanos, Gerard Urrutia-Cuchí

**Affiliations:** 1 Anaesthesiology Department, University Hospital Santa Creu i Sant Pau, 08041 Barcelona, Spain; 2 Institut d’Investigació Biomèdica Sant Pau (IIB SANT PAU), 08041 Barcelona, Spain; igichs@santpau.cat; 3 Intensive Care Units Department, University Hospital Virgen del Rocío, 41013 Sevilla, Spain; marilorincon@hotmail.com; 4 Anaesthesiology Department, Hospital Ramón y Cajal & IRy CIS, 28034 Madrid, Spain; angel.candela@salud.madrid.org; 5 Haematology Department, University Clinic of Navarra, 31008 Pamplona, Spain; mmarcos.3@unav.es; 6 Anaesthesiology Department, University Hospital Germans Trias I Pujol, 08916 Barcelona, Spain; marta_barquero@hotmail.com; 7 Department of Clinical Epidemiology and Public Health, University Hospital Santa Creu i Sant Pau, 08041 Barcelona, Spain; gurrutia@santpau.cat; 8 CIBER of Epidemiology and Public Health (CIBERESP), 28029 Madrid, Spain; 9 Blood & Tissue Bank, Hospital de la Santa Creu i Sant Pau, 08041 Barcelona, Spain; lmedina@bst.cat; 10 Blood and Tissue Bank of Catalonia, 08005 Barcelona, Spain; abosch@bst.cat; 11 Hospital Clinic de Barcelona, 08036 Barcelona, Spain; daniela.garrido.f@gmail.com; 12 Department of Biochemistry, Hospital de la Santa Creu i Sant Pau, 08025 Barcelona, Spain; jordonez1952@gmail.com; 13 Foundation for Clinical Biochemistry & Molecular Pathology, 08007 Barcelona, Spain

**Keywords:** massive hemorrhage, protocol compliance, education, mortality

## Abstract

**Background:** In 2015, Spanish scientific societies published a consensus document on managing massive hemorrhage (MH). This study aimed to evaluate the knowledge and application of the Massive Hemorrhage Protocol (MHP) among healthcare professionals and to assess whether an educational intervention could improve compliance and patient outcomes. **Methods:** A two-phase observational study was conducted in four Spanish university hospitals. In phase one, compliance with MHP recommendations was surveyed. Based on the findings, educational sessions were implemented, focusing on the least known or followed recommendations. Compliance was then reassessed. Primary outcome was adherence to MHP; secondary outcomes included morbidity and 24 h and in-hospital mortality. **Results:** The MHP was activated in 303 MH episodes, mostly of surgical (42.6%) or traumatic (25%) origin. The most followed recommendation before the intervention was protocol activation (94%), which improved to 98.3% post-intervention (*p* = 0.049). Lesser-followed recommendations such as requesting a hemorrhage lab panel and correcting hypothermia improved after intervention from 39% to 50.4% (*p* = 0.05) and 31.3% to 43.8% (*p* = 0.027), respectively. Overall compliance increased from 68% to 73% (*p* = 0.05). Mortality remained high in both phases, 24 h (25.4%) and in-hospital (42.2%). Patients who required massive transfusion had higher mortality (53.6%) than those who did not (35.9%, *p* = 0.03). Survivors had higher protocol compliance (*p* = 0.003 at 24 h; *p* = 0.049 in-hospital). **Conclusions:** Educational intervention modestly improved adherence to MHP recommendations. Higher compliance was associated with better survival outcomes, supporting the need for targeted educational strategies to enhance protocol implementation and improve care in MH cases.

## 1. Introduction

Massive hemorrhage (MH) is a life-threatening condition. Depending on the MH origin, mortality can reach up to 60% in the cases of uncontrolled MH [1,2,3,4,5]. MH mainly occurs in major surgical interventions or in traumatic, gastrointestinal or obstetric bleedings and requires immediate and multidisciplinary management to decrease its high mortality rate.

Protocols to manage MH (massive hemorrhage protocols—MHPs) have been developed to facilitate more rapid clinical decision-making and timely delivery of blood components [6]; their use has been associated with a reduction in short- and long-term mortality [3,7,8,9]. Moreover, several studies have shown that MHPs can also ameliorate the use of blood components by reducing potential wasting [10].

MHPs were first proposed by Malone in the year 2006 in a trauma setting [11] and since 2010 their use has been included in the ten mandatory safety protocols for Anesthesia and Intensive Care Departments in the Declaration of Helsinki’s guidance on patient safety [12]. However, despite the existing recommendations and the increasing use of MHPs, the mortality rate of MH patients has varied widely, suggesting that poor outcomes could be attributable to an incomplete compliance with MHP recommendations [13,14,15].

In the year 2015, three Spanish scientific societies, i.e., Anesthesiology, Intensive Care and Coronary Units and Thrombosis and Hemostasis, developed a consensus document on MH management including, among other things, the recommendation of using MHPs in patient care [16]. Additionally, the consensus recommended “to conduct information and training campaigns for the teams involved in the use of MHPs, and to periodically assess the MHP compliance and their effectiveness”. Both recommendations were based on the evidence that the use of MHPs, the training of users and surveilling the results of all these processes all help in reducing the mortality associated with MH [15].

After launching the consensus, a survey conducted by the current authors in four Spanish university hospitals detected a suboptimal knowledge of MHP recommendations, particularly those referring to the use of blood derivatives [17]. Based on the survey findings, considered as part of the present study, we aimed to evaluate whether an educational intervention could improve both compliance with MHP recommendations and patient’s outcomes.

## 2. Materials and Methods

### 2.1. Study Design

The study was observational and retrospective, conducted in four university, tertiary Spanish hospitals; the centers were selected as those having an ongoing MHP in the 5 years preceding the beginning of the study. The clinical information of adult patients requiring MHP activation was registered before (preintervention) and after (postintervention) an educational intervention on the MHP use. The preintervention group acted as a control group to analyze the changes potentially existing in the postintervention population by effect of the educational activity.

### 2.2. Preintervention

Before the educational intervention, the investigators conducted a survey based on a questionnaire with multiple-choice questions to evaluate the degree of knowledge and application of MHP recommendations among healthcare professionals who manage MH patients. The survey included several sections, such as MH logistics/organization, MHP activation/deactivation, type, preservation, prescription and administration of blood derivatives, requests for laboratory testing and personal experience of the healthcare team. The answers to the survey allowed for the detection of items less known or applied and the designing of the following steps of the study. When judged necessary, the investigators reviewed the MHPs used in the centers and updated them to the most recent evidence.

### 2.3. Educational Intervention

The intervention was developed at each hospital by the investigators of the study and consisted of a clinical session of at least one hour’s duration. The educational activity was planned to be performed only once, although the number and duration of sessions and demographics of participants were discretionally applied at each center to optimize the feasibility of the activity. The content, materials to be distributed and tools to be used in the educational activity were agreed upon and developed by the investigators of participating centers. The audience consisted of clinicians, blood bank and clinical laboratory staff and nurses involved in MHP management; the investigators encouraged the participation of physicians like anesthesiologists, surgeons, intensive care or emergency physicians in charge of the activation and management of the MHP at each center.

The sessions included a reminder of the key points of MHP activation, the presentation of survey results, with special attention to those items with a previously detected lower knowledge degree, the updates of the MHP when complete, and suggestions to improve MHP use in clinical practice. The sessions were tailored to the specific requirements of each center. To strengthen the content of the educational sessions, the investigators distributed printed checklists which included key points of MHP activation and deactivation, treatment algorithms with their triggers and targets and communication facilities to ameliorate the timely request and delivery of blood derivatives.

### 2.4. Postintervention

The effectiveness of the educational intervention was checked in two ways: by assessing the degree of compliance with MHP recommendations and comparing it with the observed preintervention and, particularly, by comparing the clinical outcomes of MH patients before and after the intervention.

To evaluate the appropriateness of MHP activation, common criteria were established; MHP activation was considered appropriate in patients whose systolic blood pressure was <80 mm Hg (attributable to bleeding) or required vasopressor agents and received ≥6 packed red blood cells (pRBC) in 24 h. In cases of intraoperative cell salvage, 300 mL of recovered blood were equal to one pRBC. Finally, massive transfusion (MT) was defined as the transfusion of ≥10 pRBCs in 24 h [2].

### 2.5. Data Acquisition

MHP use was evaluated in clinical records of all cases in which MHP activation was appropriate. Clinical data of MH patients were obtained from multiple sources (electronic records, paper charts, databases from transfusion services or viscoelastic tests (VET) results) and collected and anonymized by the investigators in a case report form (CRF). CRF data were uploaded to a centralized study database by a data manager; any missing value was considered as no compliance. Data of MHP activations were reviewed by the investigators, considering 31 months before the educational action and 21 months in the postintervention phase.

At the time of MHP activation, we registered, among other things, patient data as demographics, bleeding origin, blood pressure and heart rate and parameters from clinical laboratory and rotational thromboelastometry (ROTEM^®^), as well as previous use of anticoagulants or antiaggregant. Several clinical risk scores based on the clinical features and bleeding origins were calculated at the time of patient’s admission; in trauma patients, the ABC (Assessment of Blood Consumption) and TASH (Trauma Associated Severe Hemorrhage) scores were used as predictors of MT, and ISS (Injury Severity Score) and GCS (Glasgow Coma Score) as predictors of morbidities and mortality, and in surgical patients, the American College of Surgeons National Surgical Quality Improvement Program (ACS NSQIP) was used as a surgical risk calculator and the American Society of Anesthesiologists (ASA) score as a potential predictor of morbidity and mortality. Finally, the severity degree of patients admitted to an intensive care unit (ICU) was evaluated with the SAPS II (Simplified Acute Physiology Score) score in the first 24 h of admission, as recommended.

### 2.6. Study Endpoints

The primary endpoint was the assessment of compliance with the MHP recommendations, before and after the educational actions. Compliance was evaluated with criteria drawn from the abovementioned Spanish consensus and a previous study (Bawazeer’s criteria) [14]; a total of 13 criteria related to MHP activation, management and deactivation, ordering and use of blood derivatives and correction of some patient’s variables were evaluated.

The degree of compliance was summarized with two metrics. The first was the “Overall Episode Degree of Compliance (EDC)”, calculated by assigning a value of 1 to each criterion met and 0 when not met; the maximum attainable value was 13 and EDC was reported as median and interquartile range (IQR). The second metric was the “Overall Criterion Degree of Compliance (CDC)”, calculated from the number of YES (criterion met) responses for each criterion in all evaluated cases divided by the total number of cases; the maximum possible score per criterion was 100%. Overall, CDC was expressed as the mean value of the percentages observed for each criterion.

The secondary endpoints were the short (24 h) and long-term (in-hospital) mortality and morbidities. Mortality other than that caused by the MH was adjudicated by the investigators following the same standardized criteria and available data reports. We registered deaths due to hemorrhage, sepsis, multiorgan failure, head trauma, thrombosis, respiratory failure or other causes; death due to hemorrhage was defined as any death that occurring during the first 72 h after a MH episode and that could not be attributed to a cause other than MH. Morbidities included non-deadly sepsis or multiorgan failure, acute kidney injury, acute respiratory distress, pneumonia and thrombosis. Diagnoses were based on the most recent recommendations at the time of the study. Finally, other secondary outcomes included the lengths of hospital and intensive care unit (ICU) stays, time on mechanical ventilation, units of blood products transfused, ratios of the transfused products and use of coagulation factor concentrates. Patients were followed until their hospital discharge or in-hospital death.

### 2.7. Ethics

The study was first approved by the ethics committee of one institution (reference IIBSP-PTM-2017-16, chairperson Milagros Alonso Martinez, MD, Biomedical Research Institute Sant Pau, Barcelona, Spain) and the remaining centers subsequently adhered to the first approval, according to the legal procedure in Spain. The study followed the principles of the Declaration of Helsinki. Given the observational and retrospective nature of the study, informed consent from the patients was not required.

### 2.8. Statistical Analysis

Based on the number of MH patients attending the participating centers yearly, the study forecasted the evaluation of a minimum of 600 MH episodes during the assessment period. Only episodes in which MHP activation was deemed appropriate were included in the analysis. Variables were reported as mean ± SD or medians and interquartile range (IQR) when continuous and as percentages when categorical. Comparisons between the pre- and postintervention periods were made with Student’s *t*, Mann–Whitney’s U or Chi-square tests as appropriate. We accepted as significant a probability < 0.05 using a two-sided approach. Statistics were undertaken with the IBM-SPSS program (IBM Corp. Released 2019. IBM SPSS Statistics for Windows, Version 26.0. Armonk, NY USA: IBM Corp.).

## 3. Results

**Figure 1:**
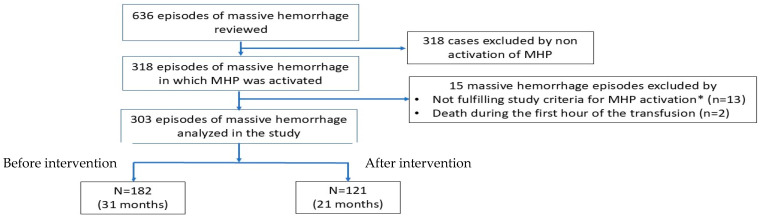
Flowchart of the study. Abbreviation: MHP: Massive hemorrhage protocol. Intervention: Educational intervention on MHP knowledge and use. * Study criteria for MHP activation: bleeding plus hypotension systolic blood pressure ≤ 80 mmHg or vasopressor requirement in patients receiving at least 6 packed red blood cells in 24 h.

We registered 636 MH episodes during the two study periods; MHPs were activated in half of cases (*n* = 318). In the remaining cases, MHPs were not activated and the cases were excluded from the study. MHPs were not activated when easy and rapid control of bleeding was foreseeable, e.g., in some surgical cases. In these patients, ROTEM^®^ or laboratory-guided therapy was used instead of using fixed ratios of blood components. After reviewing the appropriateness of MHP activation, we excluded 13 cases (5.6%) by not meeting the criteria required for MHP activation and 2 cases due to death during the first hour of attention. Accordingly, our study included 303 MH cases in which MHPs were initiated: 182 MHP cases (60.1%) during the preintervention phase (which lasted for 31 months) and 121 (39.9%) during the postintervention phase (which lasted for 21 months) (Figure 1). The number of MHP activations by month did not differ between preintervention (5.87 cases/month) and postintervention phases (5.76 cases/month).

### 3.1. Clinical and Transfusion Data

The percentage of MH inclusions varied among centers (Table 1). Sites 3 and 4 included fewer cases owing to the clinical characteristics of the population they attended to, i.e., fewer patients requiring MT compared to sites 1 and 2, and to a later beginning of their recruitment.

Patients were predominantly male (66.1%) and aged 56.5 ± 18.0 years without differences between the study phases. Traumatic and obstetric patients were significantly younger (47.5 ± 18.6 years and 30.3 ± 9.1 years, respectively,) than the remaining patients (surgery 61.6 ± 16.1 years, gastrointestinal bleeding 57.6 ± 13.0 years, other causes 63.6 ± 15.1 years; *p* = 0.0001).

Most MH episodes were associated with surgeries (42.6%), most of them cardiac (36.4%), followed by traumatic causes (25.1%), gastrointestinal bleeding (12.9%), peripartum hemorrhages (4.3%) and other causes (15.1%); hemorrhagic causes did not differ between the study phases (Table 1). Other variables also not differing between phases were previous use of antiaggregant or anticoagulant drugs—nearly one third of MH patients were on antiaggregant or anticoagulant therapies, mainly antiplatelet drugs—arterial blood pressure, heart rate, lactate and hemoglobin concentrations. Of note, before intervention, lactate and fibrinogen measures were available in only 24.7% and 53.2% of cases, respectively; after intervention, lactate requests decreased to 12.4%, whereas fibrinogen assessment was available in 58.7% of cases. Coagulation parameters were measured either at the central labs or as VET by ROTEM^®^. Interestingly, we registered a significant increase in VET use for CT-EXTEM MCF-EXTEM and MCF-FIBTEM after the intervention (from 34.1%, 33.5% and 31.9% before the educational activities to 45.4%, 44.6% and 44.6% after them, respectively; *p* ≤ 0.02 for all comparisons). Finally, there were no differences between phases in the risk scores shown in Table 1 and those calculated in those patients in which they were assessed.

Further details of administered blood components or drugs are shown in Supplementary Table S1; the use and characteristics of administered blood derivatives or active drugs did not differ between the study phases.

### 3.2. MHP Compliance and Outcomes

Table 2 summarizes compliance with the criteria chosen to evaluate the appropriateness of MHP use before and after the educational actions. After intervention, two criteria showed a statistically significant improvement. They were the percentages of MHP activations based on pre-specified indications (from 94.3% before to 98.3% after; *p* = 0.049) and the use of measures to correct or prevent hypothermia (from 31.3% before to 43.8% after; *p* = 0.029). We also observed increases, though their statistical significance was just equal to 0.05, in the compliance rates after intervention in the requesting of a hemorrhage panel from the laboratory and the fibrinogen measurement by a central laboratory or Fibtem^®^ assay (before 39.0%, after 50.4%) and in the administration of blood products to achieve a fresh frozen plasma (FFP) to red blood cell ratio ≥ 1:2 (before 50.0%, after 62.0%). VETs were more often used after intervention (in 60.5% of cases before the intervention and in 70.6% after it). Most of the remaining criteria exhibited higher compliance after intervention, but not enough of an increase to reach statistical significance.

The value of the two metrics summarizing criteria compliance, i.e., EDC and CDC, increased after the intervention, with EDC from a median and IQR of 8 (6–9) to 8 (7–9) and CDC from 68.5% to 72.9%, but improvements were only close to statistical significance (EDC: *p* = 0.053, CDC: *p* = 0.05).

### 3.3. Outcomes and Mortality

SAPS II decreased by 10% (from 52.2 ± 22.3 before to 47.1 ± 19.4 after education (*p* = 0.049)) (Table 3). The total in-hospital mortality was high but not different between intervention phases (42.3% before, 42.5% after). Most deaths occurred during the first 24 h of admission, also without significant differences between the study phases (23.6% before, 28.1% after intervention, *p* = 0.42) (Table 3). In trauma patients, mortality was 46.1%, whereas in non-trauma ones it was 42.9% (*p* = 0.07). MH was the most frequent cause of mortality (46.1%), followed by multiorgan failure (28.1%), head trauma (5.5%), sepsis (2.3%) and thrombosis (1.6%). Acute kidney injury and multiorgan failure were the most frequent morbidities observed despite their frequency, as the frequency of sepsis, pneumonia or acute respiratory distress syndromedid not vary between the before and after steps. Other outcomes, such as days on mechanical ventilation or length of ICU or in-hospital stay, did not vary after the intervention either.

Finally, improvements in compliance measured by the EDC were associated with the observed mortalities. EDC was higher in patients surviving than in those who died, as follows: in-hospital mortality [8 (7–9) vs. 8 (6–9), *p* = 0.049], 24 h mortality [8 (7–9) vs. 7 (6–9), *p* = 0.003] and mortality due to massive hemorrhage [8 (7–9) vs. 7 (6–8), *p* = 0.012] (Table 4).

### 3.4. Massive Transfusion

**Figure 2:**
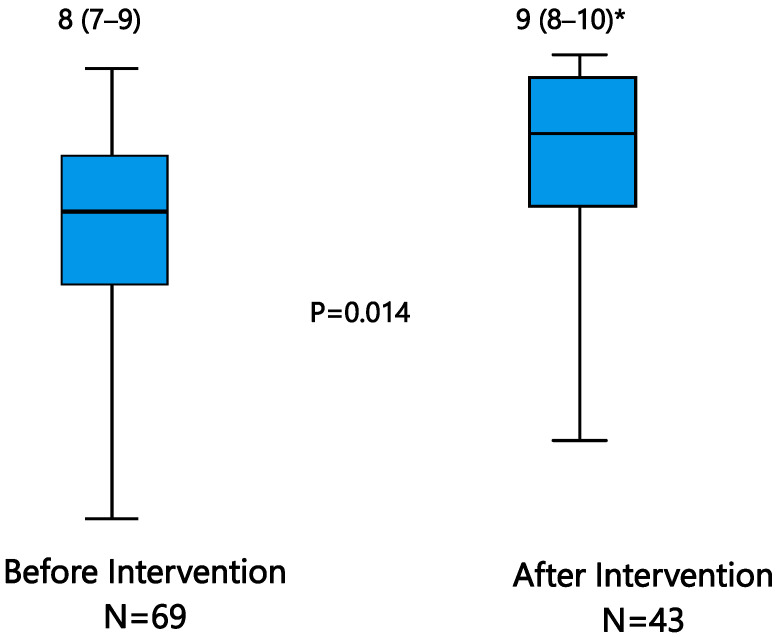
Overall episode degree of compliance before and after an educational intervention for patients receiving massive transfusion. * Data expressed as a median and (interquartile range). Only those patients with complete data of all the evaluated criteria of the massive hemorrhage protocol were included in the analysis. Massive transfusion: transfusion of 10 or more packed red blood cells in 24 h.

MT was similarly used in trauma (40.8%) and in non-trauma patients (35.7%). The percentage of MH patients receiving MT was similar in both study phases (37.9% before, 35.5% after intervention). These patients had an in-hospital mortality higher than that of patients not requiring MT (53.6% in MT, 35.9% in non-MT; *p* = 0.03), regardless of the study phase. In this patient group, the overall EDC value was significantly increased after intervention (EDC 9 (8–10)) compared to before: 8 (7–9) (*p* = 0.014) (Figure 2).

## 4. Discussion

This is the first multicenter study conducted in Spain assessing whether an educational intervention could improve compliance with MHP recommended criteria for MH patients and their outcomes. The evaluated criteria were derived from previous studies [14] and from a Spanish-based multidisciplinary consensus document about MH management [16], which has been recently updated [18]. It differed from other studies regarding analyzed populations with a unique cause of MH, e.g., trauma, as our study included a large set of patients with different MH causes.

We reviewed clinical records of MHP activations in different time periods, i.e., before and after an educational activity. However, despite the different time frames of analyses, the characteristics of cases as bleeding causes, some laboratory values and the requirements for MT or blood components administered did not differ between both phases. This suggests that differences observed in some variables before and after the intervention could be considered to be because of the educational intervention.

After the educational intervention, two MHP criteria, i.e., the percentages of MHP activations and the use of measures to prevent or correct hypothermia, increased significantly in their compliance. In fact, % of MHP activation according to prespecified criteria was the most complied-with criterion before intervention (94.3%) but it significantly improved after it (98.3%). In contrast, hypothermia correction was the most poorly complied-with criterion before (31.3%) and after (43.8%) education. Two other criteria showed compliance improvements very close to statistical significance (*p* = 0.05), i.e., the request for hemorrhage panels from the clinical laboratory, including fibrinogen measures, and the administration of blood derivatives to achieve an FFP:pRBC ratio ≥ 1:2. In a scoping review of 107 different studies using similar items to ours to measure MHP compliance [19], it was reported that the availability of lab panels was around 40%. In our study, we found a similar rate of 39% for such availability in the preintervention phase but an increase to 50% after intervention. Other studies have reported much lower rates of laboratory panel requesting, at 4% [14,20]. Regarding fibrinogen measures, we registered a significant increase of around 10% in VET measures. Since low fibrinogen concentrations are independently correlated with a higher in-hospital mortality of MH patients [21] and fibrinogen administration with mortality reduction [22], an increased availability of their measures could help to optimize MH management and the outcomes associated with the decrease in its values. On the other hand, reaching an FFP:pRBC ratio ≥ 1:2 is recommended in guidelines [23] and associated with a survival benefit in trauma patients [24,25], although such benefit in non-trauma patients is controverted [26,27]. In our study, the compliance with this recommendation increased from 50% in the preintervention to 62% after it. The failure to achieve an appropriate predefined FFP:pRBC ratio was mainly attributable to the lack of pre-thawed plasma at three of the four participating centers; in addition, any delay in MHP activation could lead to an unbalanced pRBC:FFP ratio. The early recognition of MH and rapid activation of the MHP are key to reducing mortality in MH patients [28,29]; however, an early activation of MHP has been shown to be a common reason for non-compliance with MHP, even after conducting interventions to improve it [14,15].

In our study, MHPs were activated in the first 30 min of admission in 70% of cases, with non-significant differences between phases; consequently, MHP activation was delayed more than the recommended amount in around one third of patients. A factor possibly contributing to these delays was the lack of a universal definition of major bleeding [30] and unequivocal criteria for predicting MH or MT in most clinical scenarios where it can occur [29]. Validated scores for predicting the need for MT exist only in trauma patients, but not for patients with causes other than trauma, such as those causing MH in more than 50% of our patients. Moreover, only one center of those surveyed in the study used a scoring system to predict MT in trauma patients; this was consistent with the observations in a Dutch survey including 11 trauma level 1 centers in which only one used a shock classification to activate the MHP and none followed the national guidelines [31].

Considering the described significant increases in compliance as well as the observed, though non-significant, improvements in most of the remaining recommendations, it was not surprising that metrics summarizing the overall compliance also showed increased values after education, i.e., *p* = 0.05 for CDC and 0.053 for EDC. However, some variables not directly associated with clinics, like prevention of blood product wastage or timely MHP deactivation—both highly complied with before the education (83.5% and 93.2%, respectively)—did not vary after it, and others like hypothermia management or sending hemorrhage panels to clinical laboratory, despite being improved after intervention, still showed compliance equal to or lower than 50%, mainly justified by missing digital temperature records and lactate and fibrinogen values provided by point-of-care instruments. Thus, the overall compliance improvement was diminished by variables highly complied with before the education or others with low compliance due to lack of automatic recording in medical clinical reports.

In a study conducted at a level I trauma center, it was observed that MHP compliance increased by more than 80% after educational interventions when they were delivered quarterly, and when adjustments to the protocols were carried out in “real time” [15]. Bearing in mind that our protocol’s adjustments and education activity were performed only once, our data supported the positive effect of the education on the appropriateness of MHP use in clinical practice. Future studies in populations with mixed causes of MH should investigate the influence of periodical educational activities with real-time adjustments on the MHP use and on patient outcomes and assess the feasibility and cost–benefit impacts of them.

Patients receiving MT were one third of the participants. MT is an organizational and logistical challenge for health teams and MHPs were designed to better address this challenge and particularly to optimize blood derivate use and prevent their waste. Blood wastage is not an unusual finding in centers using MT and MHP. this It has been attributed mainly to causes related to the handling of blood outside the blood bank, due to personnel shortages for the transportation of blood derivatives or inadequate temperature control when they were not transfused [32]. Blood wastage also depends on the cause of MH and the product transfused; platelets were the most wasted product, reaching more than 15% wastage in a population with mixed causes of MH [33] and in obstetric patients [34]. On the other hand, in trauma patients, the proportions of wasted platelets, pRBC or plasma were reported to be similar [35]. In our study, we found high rates of wastage prevention of 83.5% and 84% in the before and after phases, respectively, in accordance with what has been found in other studies.

Despite adequate therapy, MH is associated with high mortality. In this study, we observed an in-hospital mortality consistent with that previously reported in MH patients managed with a MHP [15,36]. Half of our registered deaths occurred in the first 24 h of admission, and this and in-hospital mortalities did not change after education regardless of the observation of a significant decrease in SAPS II score. The SAPS II could not be an optimal mortality predictor in MH due to the dynamic nature of the condition and its management, and to some inadequacy of the score in MH patients to capture the variability in mortality rates due to underlying cause of bleeding, occurrence of coagulopathy or transfusion-related complications. In fact, a study developed in ICUs of our country with similar patterns of patients revealed that SAPS II overestimate survival at the values observed in our population after the intervention but underestimate it at values found before the education [37].

In contrast with the unmodified mortality rate after the intervention, we found a statistically significant association between the increase in MHP compliance after intervention and decreased 24 h and in-hospital mortality, as well as with mortality by hemorrhage. We hypothesize that these discordant findings could be due to several factors. First, studies showing a significant mortality reduction with increasing MHP compliance included trauma patients [38], whereas our study only included 25% of trauma patients. Second, our educational intervention was conducted only once, as mentioned; probably, more continued interventions could be required to not only improve compliance but to decrease a hard outcome such as mortality of any cause. Third, our compliance assessment referred to the 13 items included in our MHP; however, some of these items are unrelated to mortality (such as preventing blood wastage or timely MHP deactivation). An MHP focused on variables more strongly related to mortality could offer different results, but the main aim of the study was to assess the MHP knowledge and use and to check whether they improved after intervention.

### Limitations and Strengths

This study had some shortcomings. Firstly, the improvements observed in the criteria’s compliance and, through them, in some clinical outcomes could be mediated through the so-called Hawthorne effect, i.e., promoting a modification of the clinical behavior of professionals involved in MHP use and management in response to their awareness that their work will be observed after the survey and the educational intervention [39]. However, quality control programs use the Hawthorne effect to improve the quality of operators based on their continuous observation. Ultimately, this promotes an overall quality improvement that in our study was evidenced by the improvement in compliance with various MHP criteria and was associated with patient’s outcomes. Secondly, we did not control for variables like some differences in clinical practices, or resources that may have changed over time or differences in the etiology of patients and involvement of professionals among the centers that also could have had potential effect on the observed results. Thirdly, the application of the educational intervention only once may limit its impact in some analyzed items in terms of improving them significantly. However, professionals attending the sessions will share the acquired knowledge with non-attendees also involved in MHP management, contributing to the further spread of the new knowledge. Fourthly, due to the retrospective design of the study, missing data may have introduced bias, especially during the pre-intervention phase. Fifthly, although the criteria to evaluate the MHPs and their application were agreed upon by the participating centers, we cannot rule out discrepancies in the registering and interpretation of some data by the investigators of each institution. Sixthly, and finally, conclusions about the cause–effect relationship of MHP compliance on mortality cannot be derived as our design only detects associations.

The main strength of the study was the large set of data analyzed, and the variety of MH causes it included. Most of the previous studies on MHP applications were developed only on MH from trauma origin. However, in our country, as in other developed countries, monographic trauma centers do not exist and our investigation included other causes besides trauma, such as surgery, peripartum or gastrointestinal bleedings, as is fully representative of our practice.

## 5. Conclusions

In this study, we developed an educational intervention on the use of MHP in patients with MH of both traumatic and non-traumatic origin. Before and after the education, we assessed compliance with MHP recommendations and how the compliance related to clinical outcomes. Overall metrics to assess compliance improved after the intervention, mainly through increases in the availability of laboratory results, the administration of blood derivatives in prespecified ratios and in hypothermia prevention/correction. The illness severity of patients assessed by SAPS II score improved after education. A higher level of compliance was significantly associated with lower immediate and in-hospital mortalities. We conclude that better compliance with MHP recommendations achieved through education is associated with improved survival rates.

## 6. Patients

Patients were not involved in the design, conduct and reporting of this study as it was not applicable to this research project.

## Figures and Tables

**Table 1:** Comparison of patient characteristics, bleeding diagnosis and clinical and laboratory findings at protocol activation before and after the educational intervention.

	Intervention Phase		*p* Value
	Before	After	
	*n* = 182	*n* = 121	
Patients by site, *n* (%)			
Site 1	60 (33.0%)	43 (35.5%)	
Site 2	91 (50.0%)	48 (39.7%)	
Site 3	17 (9.34%)	20 (16.5%)	
Site 4	14 (7.69%)	10 (8.26%)	
Male sex, *n* (%)	124 (68.1%)	76 (62.8%)	0.38
Age, years	56.0 ± 18.8	57.4 ± 16.8	0.52
Cause of hemorrhage, *n* (%)			0.15
Surgery	80 (43.9%)	49 (40.5%)	
Trauma	46 (25.3%)	30 (24.8%)	
Gastrointestinal	23 (12.6%)	16 (13.2%)	
Peripartum	11 (6.04%)	2 (1.65%)	
Other causes	22 (12.1%)	24 (19.8%)	
Previous antiaggregant use, *n* (%)	38 (20.9%)	31 (25.6%)	0.4
Previous anticoagulant use, *n* (%)	19 (10.5%)	15 (12.5%)	0.58
Systolic blood pressure, *n* (%)	155 (85.2%)	108 (89.2%)	0.8
mm Hg	79.9 ± 22.7	80.7 ± 25.9	
Diastolic blood pressure, *n* (%)	152 (83.5%)	106 (87.6%)	0.52
mm Hg	46.9 ± 13.4	48.1 ± 17.3	
Heart rate, *n* (%)	149 (81.9%)	107 (88.4%)	0.49
bpm,	101.6 ± 27.7	103.9 ± 26.1	
Lactate, *n* (%)	45 (24.7%)	15 (12.4%)	0.53
mmol/L	7.26 ± 7.45	6.17 ± 5.1	
Hemoglobin, *n* (%)	124 (68.1%)	87 (71.4%)	0.46
g/L	86.3± 30.3	83.6 ± 23.1	
INR, *n* (%)	117 (64.3%)	84 (69.4%)	0.86
ratio	1.56 ± 0.77	1.54 ± 0.69	
Partial thromboplastin time, *n* (%) ratio	114 (62.6%)	82 (67.8%)	0.27
	1.77 ± 2.00	2.15 ± 2.61	
Fibrinogen, *n* (%)	97 (53.8%)	71 (58.7%)	
g/L	2.17 ± 1.42	2.01 ± 1.23	0.44
Platelets, *n* (%)	124 (68.1%)	87 (71.9%)	0.96
×10^9^/L	1808 ± 99.0	179 ±116	
CT-EXTEM, *n* (%)	62 (34.1%)	55 (45.4%) *	0.61
sec	94.45 ± 38.6	102.45 ± 110.3	
MCF-EXTEM, *n* (%)	61 (33.5%)	54 (44.6%) *	0.76
mm	51.7 ± 12.3	51.0 ± 11.5	
MCF-FIBTEM, *n* (%)	58 (31.9%)	54 (44.6%) *	0.89
mm	11.8 ± 10.9	11.5 ± 6.94	
TASH, *n* (%)	39 (21.4%)	20 (16.5%)	0.52
score	18.3 ± 5.89	17.25 ± 6.08	
ABC, *n* (%)	39 (21.4%)	20 (16.5%)	0.24
score	2.31 ± 1.00	2.00 ± 0.79	
ISS, *n* (%)	45 (24.7%)	28 (23.1%)	0.53
score	39.4 ± 18.3	36.5 ± 19.2	
GCS score	9.13 ± 5.43	10.6 ± 4.97	0.38
Trauma patients with TBI (%)	50%	36.70%	
ACS NSQIP **, *n* (%)	134 (73.6%)	83 (68.6%)	
Serious complications (%)	21.9 ± 12.3	21.6 ± 12.3	0.84
Mortality (%)	6.3 ± 8.8	6.6 ± 9.5	0.79
ASA **, *n* (%)	155 (86.2%)	93 (76.8%)	0.06
Median (IQR)	4 (2–4)	4 (3–4)	

Quantitative variables expressed as mean ± SD otherwise indicated. * *p* < 0.02 for the difference in % of use before and after the educational intervention. ** Score measured in patients requiring surgery regardless of the cause of bleeding. Abbreviations: SD: Standard deviation, INR: International normalized ratio, CT: Clotting time, EXTEM: Tissue factor test in Rotem^®^ (Tem Innovations GmbH, Múnich, Germany), MCF: Maximum clot firmness, FIBTEM: Tissue factor test plus cytochalasin D in Rotem^®^, TASH: Trauma-associated severe hemorrhage, ABC: Assessment of Blood Consumption, ISS: Injury Severity Score, GCS: Glasgow Coma Score, TBI, Traumatic brain injury, ACS NSQIP: American College of Surgeons, National Surgical Quality Improvement Score, ASA: Physical Status Classification, IQR: Interquartile range.

**Table 2:** Compliance criteria and level of compliance before and after the intervention.

Compliance Criteria	Intervention		*p* Value	Comments
	Before *n* = 182	After *n* = 121		
1 MHP activation based on the pre-specified indications	182/193 (94.3%)	121/123 (98.3%)	0.049	Thirteen patients (5.6%) did not meet the prespecified criteria. They were not shocked, nor did they require ≥6 pRBC/24 h.
2 Timely communication with blood bank (<30 min from arrival at ED or from fulfillment of criteria to activation)	(72.8%)	(67.3%)	0.345	Time of 30 min was agreed instead of 15 min to better match MH episodes both in traumatic and non-traumatic patients.
3 Group and screen sent	(84.5%)	(87.0%)	0.615	
4 Hemorrhage panel sent to lab (ABG with lactate, CBC; INR, Fibrinogen or Fibtem and electrolytes)	(39.0%)	(50.4%)	0.05	Clauss fibrinogen and FITBEM are closely correlated, thus compliance was considered present if either of these was measured.
5 Recording of initial hemodynamic variables	(81.3%)	(86.8%)	0.269	
6 Administration of blood products to achieve ≥ 1:2 plasma to red blood cell ratio	(50.0%)	(62.0%)	0.05	Ratios were calculated at the end of the 24 h massive transfusion period.
7 Measures to correct or prevent hypothermia	(31.3%)	(43.8%)	0.029	Recorded how we warm patients or avoid heat loss.
8 Correction of acidosis	(79.1%)	(81.0%)	0.770	
9 Correction of hyperpotassemia	(77.9%)	(74.8%)	0.578	
10 Correction of hypocalcemia	(67.6%)	(75.2%)	0.159	
11 Viscoelastic tests used	(60.5%)	(70.6%)	0.083	
12 Prevent blood product wastage.	(83.5%)	(84.0%)	1.000	
13 Timely MHP deactivation	(93.2%)	(90.5%)	0.506	Within 1 h of last blood product given.
Average criterion degree of compliance (CDC)	68.5%	72.9%	0.05	
Overall episode degree of compliance (EDC), median (IQR)	8 (6–9)	8 (7–9)	0.053	

Missing data were considered as non-compliance. Abbreviations: ABG: Arterial blood gases. CBC: Cellular blood count. CI: Confidence interval. ED: Emergency department. IQR: Interquartile range. INR: International normalized ratio. MHP: Massive hemorrhage protocol. pRBC: Packed red blood cells.

**Table 3:** Morbidity and mortality outcomes at the intensive care units (ICUs).

Outcomes	Intervention Phase		*p*
	Before	After	Value
SAPS II	52.25 ±22.3	47.1 ±19.4	0.049
24 h-mortality	43 (23.6%)	34 (28.1%)	0.42
In-hospital mortality	77 (42.3%)	51 (42.5%)	0.97
Mechanical ventilation, days	2 (1–8.5)	2 (1–4)	0.21
ICU stay, days	6 (3–13)	5 (2–11)	0.13
Length of hospital stay, days	11 (1–28)	9 (1–28)	0.45

Abbreviations: SAPS II: Simplified Acute Physiology Score II. ICU: Intensive care unit.

**Table 4:** Episode Degree of Compliance (EDC) values and mortality.

24 h		In-Hospital		Mortality by Hemorrhage	
Mortality		Mortality			
Dead	Alive	Dead	Alive	Dead	Alive
7 (6–9)	8 (7–9)	8 (6–9)	8 (7–9)	7 (6–8)	8 (7–9)
*p* = 0.003		*p* = 0.049		*p* = 0.012	

EDC values expressed as median and (interquartile range).

## Data Availability

The data used in the present study are part of a larger dataset. The datasets generated and analyzed during the current study are available and can be supplied by the corresponding authors upon reasonable request. The data not used for this manuscript will be employed in future manuscripts.

## Supplementary Materials

The following supporting information can be downloaded at https://www.mdpi.com/article/10.3390/jcm14134632/s1, Table S1: Transfusion data.
