# Supplementary material for: Improving the Compliance of Massive Hemorrhage Protocols Through Education Is Associated with Patient Survival
Source: J Clin Med. 2025 Jun 30;14(13):4632. doi: 10.3390/jcm14134632 (PMC12250384; doi:10.3390/jcm14134632)
Supplement: Supplementary file 1 [file jcm-14-04632-s001.zip › jcm-3674614-supplementary.pdf]

Supplementary Table S1. Transfusion data.

	Intervention phase		p-value
	Before (n=182)	After (n=121)	
Massive transfusion % (n/total)<br>- in trauma patients<br>- in non-trauma patients	37.9%<br>41.3% (19/46)<br>36.8% (50/136)	35.5%<br>40.0% (12/30)<br>34.1% (31/91)	NS*
pRBC units	9.7 ± 6.6	9.7 ± 7.4	0.75
FFP units	4.7 ± 4.9	5.3 ± 5.5	0.45
Platelets pools	1.7 ± 1.8	1.8 ± 2.2	0.74
Cryo units	0.08 ± 0.7	0.3 ± 1.7	0.31
FFP to RBC ratio	0.53 ± 0.3	0.59 ± 0.36	0.16
Platelet-to-RBC ratio	0.21 ± 0.14	0.22 ± 0.12	0.98
Fibrinogen concentrate,	113 (62.1%)	82 (67.8%)	0.3
administered dose, grams	4.3 ± 2.7	4.3 ± 3.4	0.38
Prothrombin complex concentrate,	29 (15.9%)	23 (19%)	0.5
administered dose, IU	1483 ± 829	1757 ± 1257	0.37
Factor VIIa	5 (2.7%)	5 (4.1%)	0.52
Desmopressin	7 (3.8%)	4 (3.3%)	1.00
Tranexamic acid	91 (50%)	63 (52.1%)	0.72
administered dose, grams	1.57 ± 0.82	1.70 ± 0.94	0.48

Data expressed as the number (%), mean ± SD or median (interquartile range) unless otherwise indicated.

*NS = Non-significant difference for all comparisons.

Abbreviations: pRBC: packed red blood cells (RBC), FFP: fresh frozen plasma, Cryo: cryoprecipitate, IU: international units.
